# Comparison of dolutegravir and efavirenz on depression, anxiety and sleep disorders in pregnant and postpartum women living with HIV

**DOI:** 10.1097/QAD.0000000000003852

**Published:** 2024-03-06

**Authors:** Lena van der Wekken-Pas, Sylvia Nassiwa, Thokozile Malaba, Mohammed Lamorde, Landon Myer, Catriona Waitt, Helen Reynolds, Saye Khoo, Nengjie He, Liesbeth van Leeuwen, David Burger, Duolao Wang, Angela Colbers

**Affiliations:** a Radboud University Medical Center, Department of Pharmacy, Radboud Institute for Medical Innovations (RIMI), Nijmegen, the Netherlands; b Research Department, Infectious Diseases Institute, Makerere University, Kampala, Uganda; c School of Public Health & Family Medicine, University of Cape Town, Cape Town, South Africa; d Department of Pharmacology & Therapeutics, University of Liverpool; e Department of Clinical Sciences, Liverpool School of Tropical Medicine, Liverpool, UK; f Department of Obstetrics and Gynecology, Amsterdam UMC location University of Amsterdam, Amsterdam, Netherlands.

**Keywords:** anxiety, depression, dolutegravir, efavirenz, HIV, neuropsychiatric side effects, postpartum, pregnancy, sleep disorders

## Abstract

**Background:**

Both dolutegravir and efavirenz are known to be effective in pregnancy and postpartum to prevent vertical transmission of HIV and to maintain maternal health. Both drugs have also been associated with neuropsychiatric symptoms. To what extent, these symptoms occur in pregnant and postpartum women, however, is not yet known.

**Methods:**

This was a secondary analysis of the DolPHIN2 study, a multicentre randomized trial among women presenting late in pregnancy with untreated HIV – who received either a dolutegravir-containing or efavirenz-containing regimen. Longitudinal measures of depression, anxiety and sleep quality were analysed during pregnancy and up to 48 weeks postpartum.

**Results:**

Among 268 women, median (IQR) Edinburgh Post Natal Depression Score (EPDS) scores were 8 (3–11) and highest at enrolment. In the dolutegravir and efavirenz arm, respectively, 23.7 and 25.6% had an EPDS score above 9, indicating possible or probable depression. Abnormal Hospital Anxiety Depression scores (HADS) (above 11) were seen at least once during follow-up in 42 of patients (15.7%), although no differences were seen between treatment arms. No association was found between EPDS, suicidality and HADS scores and the assigned regimen (*P* = 0.93, 0.97 and 0.18 respectively). Median (IQR) Pittsburgh Sleep Quality index (PSQI) scores for dolutegravir and efavirenz were 6 (5–7) and 5 (5–6.5), respectively, *P* = 0.70.

**Conclusion:**

No statistically significant differences were observed between efavirenz-containing or dolutegravir-containing regimens. Rates of depression were high, but decreased over the course of time and confirm the need for psychological support after initial HIV diagnosis in pregnancy.

## Introduction

The risk of developing neuropsychiatric symptoms during pregnancy is high [1], especially following a new diagnosis of HIV. Additionally, components of combination antiretroviral therapy (cART) such as the nonnucleoside reverse transcriptase inhibitor (NNRTIs) efavirenz (EFV) and integrase strand-transfer inhibitor (INSTIs) dolutegravir (DTG) have also been associated with depression. Discontinuation rates due to adverse events in EFV-treated patients were higher compared with those treated with DTG in a large network-meta-analysis [2]. When looking specifically at depression or anxiety, the rates differed between DTG vs. EFV-treated patients (7 vs. 14% for depression and 9 vs. 12%, respectively, for anxiety). No differences were seen in the rates of insomnia [3]. In contrast, the SPRING trial showed lower rates of insomnia in DTG (2%)-treated patients, than in EFV-treated patients (10%) [4]. Also, abnormal dreams were more often reported in the EFV arm (6 vs. <1%) [5]. A cohort study of patients on DTG by de Boer *et al.*[6] reported that 13.7% of their patients stopped using DTG because of drug intolerances, of which 5.6% were sleep disturbances and 2.5% were psychological symptoms.

Unfortunately, no data exists on the rate of these side effects in pregnant and postpartum women, because aforementioned studies were performed in a predominantly male population. During pregnancy and postpartum, many things change in a woman's life. Due to hormonal, social and physical changes, the risk of anxiety and depression is increased. This might lead to sleeping disorders. Vice versa, sleeping disorders (mainly insomnia) have been associated with postnatal depression, anxiety and has been reported to diminish mother to child bonding [7]. Mental issues impact quality of life in a negative way and may hamper to function in day-to-day life [8].

Treatment interruption or cessation of cART is not feasible during pregnancy and lactation, because cART is necessary to preserve maternal health, but even more to prevent vertical transmission [9–11]. Both DTG and EFV have shown to be highly effective, even when started in late pregnancy [12,13]. There is a need to understand the contribution of drug-associated neuropsychiatric toxicity to the overall risk of depression in pregnancy. Therefore, the aim of this study was to compare the rate of depression, anxiety and sleeping disorders in pregnant and postpartum women using either a DTG-containing or EFV-containing regimen.

## Methods

### Study design

This was a sub-study of Dolphin2 (NCT03249181), a multicentre study in which women with HIV presenting late in pregnancy were randomized to receive a DTG-based or EFV-based regimen. Efficacy was assessed in terms of viral load suppression and mother-to-child transmission rates. Methods and results have been published elsewhere [12]. The current study focusses on the results of questionnaires obtained within this Dolphin-2 trial.

Participants were eligible for participation if they were 18 years or older, pregnant with an estimated gestation of at least 28 weeks and were HIV-positive and treatment-naive. Approval was obtained from the ethical review committees of South-Africa, UK and Uganda. Participants gave written informed consent. The study was conducted in South Africa and Uganda between 2018 and 2019.

### Procedures

The Edinburgh Postnatal Depression Scale (EPDS) was used to assess the rate of depressive symptoms. This tool was developed and validated to screen for postnatal depression. It consists of 10 questions with a 0–3 scoring system [14]. Total scores of 9–11 are associated with possible depression, 12–13 with fairly high possibility of depression and scores greater than14 with a probable depression. Moreover, a positive score on question 10 is associated with increased suicidality.

The Hospital Anxiety and Depression Score (HADS) was used for additional assessment of depressive symptoms and for the screening of anxiety symptoms [15]. This questionnaire consists of seven questions regarding depression symptoms and seven questions regarding anxiety-related symptoms. Each question can be scored 0–3. Scores higher than 11 are deemed to be abnormal and below 7 normal. In-between scores are regarded to be borderline.

EPDS and HADS questionnaires were completed at screening, 1 and 4 weeks thereafter, at 36 weeks of gestation and 6, 12, 24 and 48 weeks postpartum.

Sleep quality was assessed using the Pittsburgh Sleeping Quality Index (PSQI) at 24 weeks postpartum. In this questionnaire, seven different components are scored to form a total score, of which higher scores are associated with poorer sleep quality and scores greater than 5 have best sensitivity and specificity to discriminate between poor and good sleep quality [16]. This screening tool is validated in pregnant and postpartum women and is widely used in studies assessing sleep quality in this population [17].

### Statistical analysis

No formal power calculation was performed for this sub study, because the sample size was based on the primary endpoints of the original study. Analysis was performed along intention-to-treat principles.

To compare differences in sleeping quality in patients who received a DTG-containing or EFV-containing regimen Wilcoxon rank sum test was used. Linear mixed models were used to see whether the treatment regimen was associated with higher EPDS or HADS scores over time. To examine whether one of the regimens was associated with higher proportion of women giving a positive answer on EPDS question 10 (suicidality) a generalized linear mixed model (GLMM) was used for binomial variables.

Total scores on specific questionnaires were used as dependent variables, regimen and visit number and interaction between time and treatment as fixed effects, baseline measurements as covariates and subject as a random effect. Between-group difference at each visit as well as within-group difference at each visit were derived. Missing observations were not imputed but regarded as missing completely. Socio-economic factors that might influence the scores on questionnaires, were also added to the model as fixed effects [educational level, employment, marital status and study site (South Africa versus Uganda; see Table 1]. Only factors that significantly improved the model – according the lowest Akaike information criterion (AIC) – were used in the final analysis. The GLMM was constructed in a similar matter, with a binomial outcome (score of 0 or higher) as the dependent variable.

**Table 1:** Baseline characteristics.

	Dolutegravir (*n* = 135)	Efavirenz (*n* = 133)
Age (year), mean ± SD	29.5 ± 5.3	29.5 ± 5.3
Site [*n* (%)] ^∗^		
Uganda	70 (51.9%)	69 (51.9%)
South Africa	65 (48.1%)	64 (48.1%)
Education level [*n* (%)]^∗^		
Primary	21 (19.6%)	30 (28.0%)
Secondary	78 (72.9%)	64 (59.8%)
Tertiary/vocational	3 (2.8%)	6 (5.6%)
Higher/university	5 (4.7%)	7 (6.5%)
Employment [*n* (%)]		
Unemployed	63 (52.5%)	65 (55.1%)
Employed	57 (47.8%)	53 (44.9%)
Marital status [*n* (%)]		
Married	19 (17.9%)	12 (11.4%)
Unmarried	92 (82.1%)	92 (88.6%)

Values depicted with asterisk (∗) significantly improved linear mixed model and were therefore included. SD, standard deviation.

Statistical analysis was performed using R and RStudio (version 4.1.3, 2022-03-10).

## Results

A total of 268 women participated in this trial. At inclusion, the mean age of participants was 27.7 ± 5.2 years, median estimated gestational age 31 (29–34) week, median viral load 4.4 (3.8–4.8) log_10_ copies/ml and median CD4^+^ cell count 446 (296–633). A full description of the demographics of these participants from the original Dolphin-2 study is published elsewhere [10]. Overall, no differences at baseline were detected between DTG-containing or EFV-containing regimens in terms of risk of depression, anxiety or sleeping disorders.

### Depression and anxiety

**Fig. 1:**
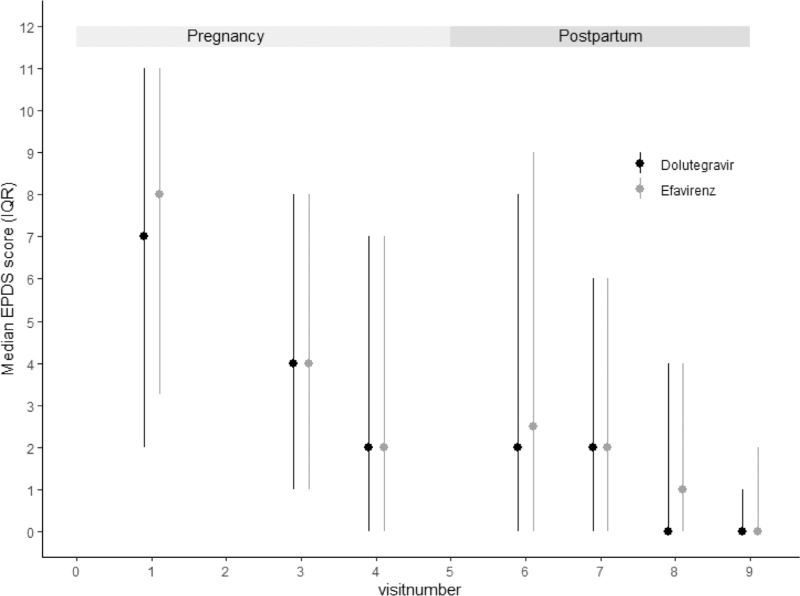
Median (interquartile range) Edinburgh Post Natal depression score per visit divided by treatment.

**Fig. 2:**
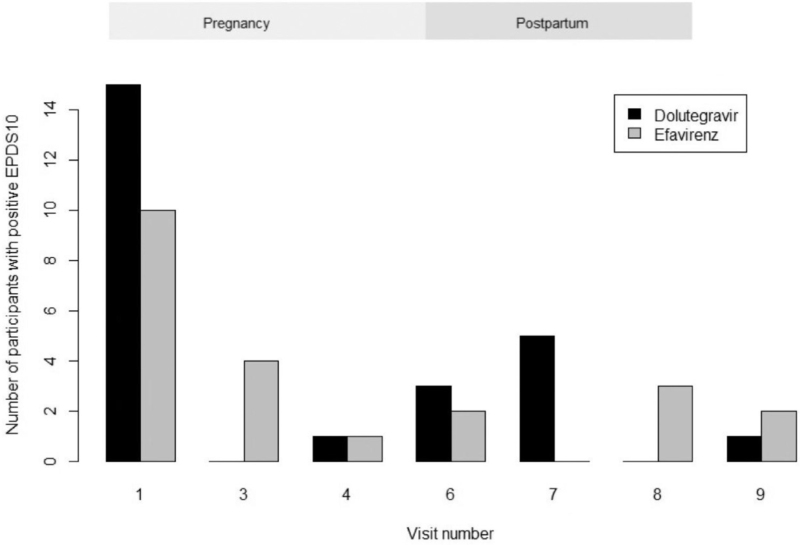
Median (interquartile range) HADS scores over time, divided per regimen.

**Fig. 3:**
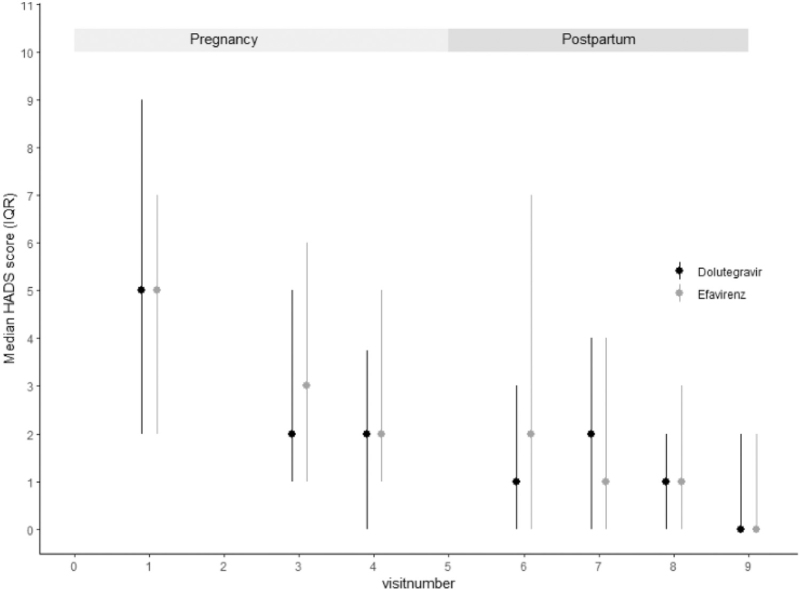
Number of positive answers on question 10 of Edinburgh Post Natal depression score questionnaire divided per regimen.

EPDS and HADS scores over time are presented in Figs. 1 and 2. Median (IQR) EPDS scores were highest at time of inclusion when participants had initially been diagnosed with HIV [8 (3–11)], compared with 2 (0–6) at 12 weeks postpartum, 0 (0–4) at 24 weeks postpartum, and 0 (0–1.5) at 48 weeks postpartum. No association was found between the treatment arm and scores on these questionnaires or specifically on question 10 of the EPDS (Table 2 and Fig. 3). Mean scores and slopes for treatment effects at individual time points are summarized in Table S1.

Visit number 1 corresponds with inclusion, 2 with 1 week after inclusion, 3 with 4 weeks after inclusion, 4 with 36 weeks of gestation, 5 till 9 with six, 12, 24 and 48 and 72 weeks postpartum.

Visit number 1 corresponds with inclusion, 2 with 1 week after inclusion, 3 with 4 weeks after inclusion, 4 with 36 weeks of gestation, 5 with delivery and 6 till 9 with six, 12, 24 and 48 weeks postpartum.

**Table 2:** Results from linear mixed model analyses of clinical outcomes.

Questionnaire	*B*	95% CI	*P* value
EPDS	−0.05	−1.15 to 1.05	0.93
EPDS10	−0.03	−1.73 to 1.82	0.97
HADStotal	0.84	−0.39 to 2.07	0.18
HADS-a	0.69	−0.05 to 1.44	0.07
HADS-d	−0.10	−0.56 to 0.37	0.69

*B* corresponds with effect of regimen on scores on questionnaires. CI, confidence interval; EPDS, Edinburgh Post Natal Depression Score; HADS, Hospital Anxiety Depression scores.

These scores are associated with suicidality. Visit number 1 corresponds with inclusion, 2 with 1 week after inclusion, 3 with 4 weeks after inclusion, 4 with 36 weeks of gestation, 5 till 9 with six, 12, 24 and 48 and 72 weeks postpartum.

From all the retrieved EPDS scores, around a quarter (DTG 23.7% vs. EFV 25.6%) were at least 9, which indicates a possible to probable depression. Scores of 14 or higher were seen in 8.1 and 8.0% of the questionnaires from participants assigned to DTG and EFV, respectively. At individual patient level, 128 (47.8%) had a score at least 9 at least once during follow-up period. Of these patients, 65 (24.3%) were assigned to EFV and 63 (23.5%) to DTG. Scores at least 14 were seen in 34 (12.7%) patients, from whom 15 (5.6%) on EFV and 19 on DTG (7.1%). Highest scores were seen at inclusion, when participants were recently diagnosed with HIV. However, 39 patients had an increase in EPDS score later during follow-up, of which 24 had scores associated with possible depression and 8 had scores of 14 corresponding with a probable depression. Of these patients, four were assigned to DTG and four to EFV (Figure S1).

A similar pattern was seen for the HADS scores. Median (IQR) HADS was highest at inclusion 5 (2–9) and decreased over time to a median (IQR) of 0 (0–2) at 48 postpartum. Abnormal scores (above 11) were seen in 49 of 834 (5.9%) of the questionnaires in the DTG arm and 67 of 826 (8.1%) in the EFV arm. Total scores greater than 11 were seen at least once during follow-up in 42 of patients (15.7%), from whom 21 (7.8%) were using DTG. When concentrating on the subscales of anxiety or depression of the questionnaires the following median scores (IQR) were observed: 1 (0–3.0) in both DTG-treated and EFV-treated patients on anxiety subscale and 0 (0–2.0) for both treatment groups on the depression subscale. 3.4% of patients had a HADS-a score above 11, associated with anxiety, from whom 4 (1.5%) used EFV and 5 (1.9%) DTG. Median scores (IQR) for the depression subscale of the HADS were 0 (0–0.2) for both DTG and EFV treated patients. Only one patient had scores greater than 11; she was assigned to EFV. All scores and model outcomes are summarized in Supplementary Data.

### Sleeping quality

No differences were seen in median (IQR) PSQI scores for DTG and EFV; 6 (5–7) and 5 (5–6.5) respectively, *P* = 0.714. In total, 46.5% of participants experienced poor sleeping quality. Moreover, no differences were seen in odds ratios for poor sleep quality (PSQI > 5) between patients receiving DTG or EFV (OR 1.24, 95% CI 0.54–2.90).

## Discussion

Our study did not find any differences between EFV-treated and DTG-treated participants in terms of sleeping quality, rate of depression or anxiety, which is partly in accordance with previous research. Ochanda *et al.*[1] evaluated quality of life within the same patient population as the present study and did not find any differences on the mental component of the Medical Outcome Study – HIV health survey (MOS-HIV) either. Other studies did find higher rates of depression and anxiety [2] and sleep disturbances in patients using DTG compared with other regimens [3,4]. Also, analysis of participant-level data from four AIDS Clinical trial group studies in treatment-naive patients showed an association between the use of EFV and suicide events (ideation and attempts/completed suicides) [5]. However, another study with data from the D:A:D cohort did not find such an association [6].

It is difficult to compare studies on neuropsychiatric adverse events, because various screening tools and different patient populations were used to establish an association. MOS-HIV [1], Centre for Epidemiologic studies Depression scale (CES-D) [7] and self-reported symptoms [4,8] are instruments with which these symptoms were assessed. This might have led to an under- or overestimation of the actual rate. Furthermore, analysing treatment-naïve or experienced populations might have introduced confounding by indication, further complicating the interpretation of the results. Finally, the sensitivity and specificity of screening tools vary by populations they are used in. In our study, a different rate of depression symptoms was noted in the EPDS and HADS questionnaires. In a Dutch cohort of pregnant women the HADS-a, EPDS and other screening tools were assessed and showed a low performance of the HADS-a [18]. Moreover, psychometric analysis of the HADS score, showed poor test-retest reliability in a pregnant cohort. The authors recommended the use of other screening tools than HADS in a pregnant population, because this tool lacks robustness [19].

Overall, depression and poor sleep quality were more prevalent in our population in comparison to others, whereas anxiety was reported less often. Depending on which definition was used, 8–25.6% of participants in this study had a possible to probable depression, which is more than the previously reported (2.5–6%) [8,9]. In the general population, the rate of depression during pregnancy and postpartum is 10–16 and 10–15%, respectively [20]. The rate of depressive symptoms in our study was notably high at start of the study and decreased over time. This is comparable to the study of Knettel *et al.*[21] who focused on suicidal ideation. Probably, the fact that participants in our study had just received word on their diagnosis of HIV is a greater contributing factor for developing these symptoms than the treatment is. It is reassuring that after delivery, no increase was seen in the rate of depression, which underlines the safety of ART postpartum. In our study, 3.4% of participants had HADS-a scores associated with anxiety, whereas Walmsley *et al.*[3] reported 3 vs. 6% in DTG and EFV-treated patients, respectively. Almost half of participants in the present study experienced poor sleep, although no differences were seen between the two assigned regimens. Sleep disturbances in our study were more prevalent than in previous studies where it was less than 6% [4,5], which might be explained by the fact that our cohort consists of mothers with young children, leading to interrupted and less sleep. This is confirmed by a systematic review and meta-analysis, in which prevalence of poor sleep quality in perinatal women was 54.2% [17].

Our study has several strengths. First, the randomized design of the original trial from which the surveys were obtained, allows for a comparison of side effects between two treatment arms. Previous studies [6] were not randomized and a control group was lacking, which might have led to channelling bias. Second, the longitudinal design and inclusion of socio-economic factors in this study are important, because many neuropsychiatric syndromes fluctuate over time and have a multifactorial cause. Williams *et al.* noted the importance of taking socio-economic factors and longitudinal gathered data into account when analysing clinical data on such disorders [7]. Their study on several ART and depression, for example, showed no association on items on CES-D for DTG and only in a positive way for EFV. Another strength is the use of clinically validated tools to assess the risk of depression, anxiety and sleeping quality, which makes it easier for future research to compare results. The use of self-reported symptoms could introduce information bias and is, therefore, better avoided.

This study is limited by the fact that a majority of participants was breastfeeding their infant, and breastfeeding is known to reduce the risk of postnatal depression [10], which makes it difficult to extrapolate our findings to high-income and middle-income countries where guidelines currently advise against breastfeeding. Here, the absence of drug-effect on the development of neuropsychiatric symptoms cannot be excluded.

According to the Department of Health & Human Services (DHHS), closer surveillance is needed for depression and suicidality in pregnant and postpartum women. Indeed, our study shows high rates of depression and poor sleep quality, but these were not different between regimens. These effects are better explained by the recent diagnosis of HIV and transition into motherhood, which comes with its own psychological challenges. It underlines the need for peripartum support to assure drug adherence and timely implementation of psychological care, especially in women who are diagnosed during pregnancy.

In conclusion, no differences were observed in rates of depression, anxiety or sleeping disorders in women diagnosed with HIV late in pregnancy treated with EFV-containing or DTG-containing regimens throughout pregnancy and after delivery.

## Acknowledgements

Funding: the DolPHIN-2 Study was funded by Unitaid (grant number 2016-08-UoL). Dolutegravir was donated by ViiV Healthcare. The funder did not take part in the design and execution of the study or the writing of the manuscript. We acknowledge the invaluable and generous contributions from all study pa00rticipants, and from staff across health facilities who supported recruitment into our study.

Contributions: L.W. wrote the manuscript. L.W., A.C., D.W. and D.B. designed the study. S.N., T.M., M.L., L.M., C.W., H.R., S.K. and N.H. were responsible for study execution and data-gathering. A.C. was responsible for monitoring. L.W. and D.W. analysed the data. A.C., D.B., D.W. and E.L. reviewed first drafts of manuscript, all other authors read and reviewed last draft of manuscript.

### Conflicts of interest

A.C. received research grants from ViiV Healthcare, Gilead, Merck, all paid to the institution. There are no conflicts of interest for the remaining authors.

## Supplementary Material

Supplemental Digital Content
