# Supplementary material for: Comparison of dolutegravir and efavirenz on depression, anxiety and sleep disorders in pregnant and postpartum women living with HIV
Source: AIDS. 2024 Mar 6;38(7):975–81. doi: 10.1097/QAD.0000000000003852 (PMC11064908; doi:10.1097/QAD.0000000000003852)
Supplement: Supplemental Digital Content [file aids-38-0975-s001.docx]

**Supplementary material**


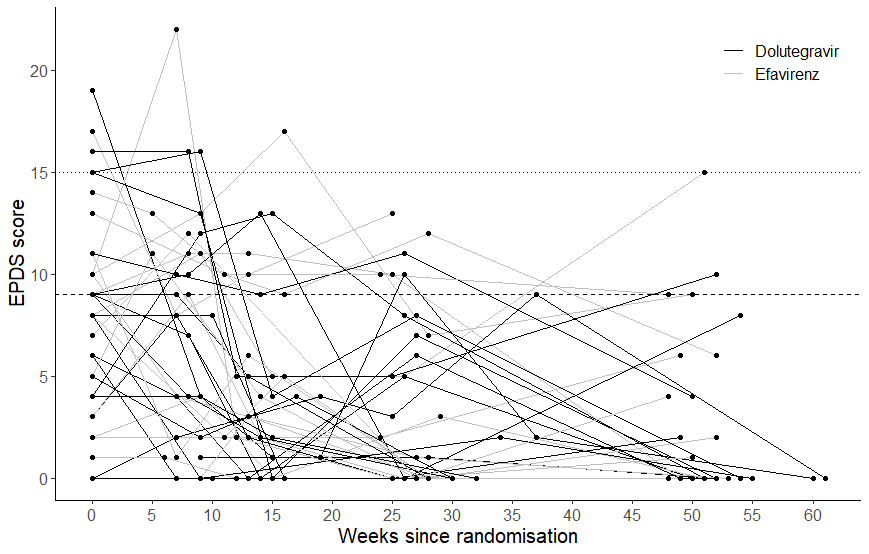


*Figure S1* *Course of EPDS scores in patients with higher scores during follow up compared to initiation of study drug. Dashed lines indicate scores associated with possible depression, dotted lines with probable depression*

*Table S1 Outcomes of (G)LMM. As adding an interaction to the GLMM did not improve the model and prohibited converging, no accurate between—and within group differences per visit could be generated.*

| Questionnaire | Visit | Mean ± SD score for DTG treated patients or in case of EPDS 10: n of individuals with positive answer (%) | Mean ± SD score for EFV treated patients or in case of EPDS 10: n of individuals with positive answer (%) | Within group difference, visit compared to baseline (95% CI, p-value) | Between group differences, visit compared to baseline (95% CI, p-value) |
| --- | --- | --- | --- | --- | --- |
| EPDS | Baseline | 7.21 ± 5.41 | 7.80 ± 5.06 | NA | NA |
|  | 4 weeks after inclusion | 5.15 ± 5.32 | 5.16 ± 5.23 | 1.84 (0.30 – 3.38, 0.02) | -0.05 (-1.15 – 1.05, 0.93) |
|  | 36 weeks of gestation | 4.43 ± 5.73 | 4.32 ±5.18 | 0.28 (-1.15 – 1.05, 0.62) | -1.04 (-2.58 – 0.50, 0.62) |
|  | 6 weeks post partum | 4.25 ± 5.18 | 4.55 ± 5.31 | -0.89 (-1.76 - - 0.03, 0.04) | -0.21 (-1.43 – 1.01, 0.19) |
|  | 12 weeks post partum | 3.76 ± 4.55 | 3.64 ± 4.45 | -1.40 (-2.26 - - 0.53, 0.00) | -0.56 (-1.78 – 0.65, 0.37) |
|  | 24 weeks post partum | 2.41 ± 3.55 | 2.95 ± 4.00 | -2.54 (-3.41 - - 1.68, 0.00) | -0.07 (-1.28 – 1.14, 0.91) |
|  | 48 weeks post partum | 1.31 ± 2.93 | 1.75 ± 3.1 | -3.78 (-4.64 - - 2.92 ,0.00) | 0.10 (-1.12 – 1.32, 0.87) |
| EPDS10 | Baseline | 15 (9.3%) | 10 (6.1%) | NA | NA |
|  | 4 weeks after inclusion | 0 (0%) | 4 (3.0%) | NA | NA |
|  | 36 weeks of gestation | 1 (1.4%) | 1 (1.4%) | NA | NA |
|  | 6 weeks post partum | 3 (1.9%) | 2 (1.3%) | NA | NA |
|  | 12 weeks post partum | 5 (3.2%) | 0 (0%) | NA | NA |
|  | 24 weeks post partum | 0 (0%) | 3 (1.9%) | NA | NA |
|  | 48 weeks post partum | 1 (0.6%) | 2 (1.3%) | NA | NA |
| HADSa | Baseline | 3.76 ± 3.36 | 3.85 ± 3.31 | NA | NA |
|  | 4 weeks after inclusion | 2.61 ± 3.16 | 3.30 ± 3.05 | 0.82 (0.02 – 1.43, 0.01) | 0.45 (-0.32 – 1.21, 0.25) |
|  | 36 weeks of gestation | 2.46 ± 2.87 | 2.42 ± 2.81 | 0.55 (-0.20 – 1.29, 0.15) | -0.28 (-1.32 – 0.77, 0.61) |
|  | 6 weeks post partum | 2.02 ± 2.80 | 2.85 ± 3.62 | -0.26 (-0.86 – 0.34, 0.15) | 0.61 (-0.25 – 1.47, 0.17) |
|  | 12 weeks post partum | 1.94 ± 2.26 | 1.89 ± 2.55 | -0.20 (-0.79 – 0.38, 0.50) | -0.66 (-1.52 – 0.19, 0.13) |
|  | 24 weeks post partum | 1.07 ± 1.57 | 1.52 ± 2.23 | -1.23 (-1.82 - - 0.64, 0.00) | -0.43 (-1.28 – 0.42, 0.32) |
|  | 48 weeks post partum | 0.92 ± 1.83 | 1.38 ± 2.07 | -1.41 (-2.00 - -0.81, 0.00) | -0.44 (-1.29 – 0.42, 0.32) |
| HADSd | Baseline | 2.71 ± 2.31 | 2.83 ± 2.54 | NA | NA |
|  | 4 weeks after inclusion | 1.68 ± 1.85 | 2.08 ± 2.50 | 0.52 (-0.05 – 1.08, 0.08) | 0.55 (-0.15 – 1.24, 0.13) |
|  | 36 weeks of gestation | 1.50 ± 2.15 | 1.67 ± 2.15 | -0.22 (-0.81 – 0.36, 0.46) | -0.24 (-1.07 – 0.58, 0.57) |
|  | 6 weeks post partum | 1.27 ± 2.09 | 1.56 ± 2.04 | -0.33 (-0.79 – 0.14, 0.17) | -0.07 (-0.73, 0.83) |
|  | 12 weeks post partum | 1.13 ± 1.80 | 1.17 ± 1.82 | -0.42 (-0.88 – 0.14, 0.07) | -0.40 (-1.05 – 0.25, 0.23) |
|  | 24 weeks post partum | 0.63 ± 1.28 | 0.82 ± 0.67 | -0.97 (-1.43 - -0.51, 0.00) | --0.42 (-1.07 – 0.23, 0.21) |
|  | 48 weeks post partum | 0.33 ± 0.78 | 0.48 ± 1.12 | -1.24 (-1.71 - - 0.79, 0.00) | -0.38 (-1.04 – 0.27 ,0.26) |
